# DEPTOR is a direct p53 target that suppresses cell growth and chemosensitivity

**DOI:** 10.1038/s41419-020-03185-3

**Published:** 2020-11-12

**Authors:** Danrui Cui, Xiaoqing Dai, Longyuan Gong, Xiaoyu Chen, Linchen Wang, Xiufang Xiong, Yongchao Zhao

**Affiliations:** 1 Key Laboratory of Combined Multi-Organ Transplantation, Ministry of Public Health, the First Affiliated Hospital, Zhejiang University School of Medicine, Hangzhou, China; 2 Institute of Translational Medicine, Zhejiang University School of Medicine, Hangzhou, China; 3 Cancer Institute of the Second Affiliated Hospital, Zhejiang University School of Medicine, Hangzhou, China

**Keywords:** TOR signalling, Transcriptional regulatory elements

## Abstract

DEP-domain containing mTOR-interacting protein (DEPTOR), a natural mTOR inhibitor, has essential roles in several processes, including cell growth, metabolism, apoptosis, and immunity. DEPTOR expression has been shown to be diversely controlled at transcriptional levels in cell- and context-specific manners. However, whether there is a general mechanism for the regulation of DEPTOR expression remains largely unknown. Here, we report that DEPTOR is a downstream target of the tumor suppressor, p53, whose activity is positively correlated with DEPTOR expression both in vitro in cell cultures and in vivo in mouse tissues. Mechanistically, p53 directly binds to the *DEPTOR* promoter and transactivates its expression. Depletion of the p53-binding site on the *DEPTOR* promoter by CRISPR-Cas9 technology decreases DEPTOR expression and promotes cell proliferation and survival by activating AKT signaling. Importantly, inhibition of AKT by small molecular inhibitors or genetic knockdown abrogates the induction of cell growth and survival induced by deletion of the p53-binding region on the *DEPTOR* promoter. Furthermore, p53, upon activation by the genotoxic agent doxorubicin, induces DEPTOR expression, leading to cancer cell resistance to doxorubicin. Together, DEPTOR is a direct p53 downstream target and contributes to p53-mediated inhibition of cell proliferation, survival, and chemosensitivity.

## Introduction

Mammalian target of rapamycin (mTOR), an evolutionarily conserved serine/threonine protein kinase, has pivotal roles in the coordination of cell responses to various stimuli and serves as a central regulator of cell proliferation, survival, and autophagy^1–3^. In mammalian cells, mTOR forms two distinct complexes, mTORC1 (consisting of mTOR, raptor, PRAS40, and GβL) and mTORC2 (consisting of mTOR, rictor, mSin1, protor, and GβL). It is well known that mTORC1 is mainly involved in the regulation of protein translation, cell growth, and proliferation by phosphorylating S6K1 and 4E-BP1, whereas mTORC2 regulates cell survival by directly phosphorylating and activating AKT^1,2^. DEP-domain containing mTOR-interacting protein (DEPTOR) inhibits the kinase activity of both mTORC1 and mTORC2 by directly binding mTOR^4^. Given that mTOR signaling is frequently activated in many human cancers, DEPTOR usually acts as a tumor suppressor, by inhibiting mTOR, to suppress cell growth, and survival via inactivation of AKT^5,6^. However, in specific cancer types, such as multiple myeloma and T-cell acute lymphoblastic leukemia, DEPTOR exhibits oncogenic properties by activating AKT to alleviate the negative feedback effect of the mTORC1 substrate, S6K1, on IRS1/PI3K signaling^7^. Given that DEPTOR expression is highly cell-specific and context-dependent^7^, its transcriptional regulation likely contributes to the complexity of its roles in tumorigenesis. Thus, it is important to determine whether the expression of DEPTOR can be regulated under both normal and stressed conditions.

In response to various cellular stresses, including DNA damage, oncogenic activation, hypoxia, and ribosomal stress, the tumor suppressor, p53, is activated to transcriptionally activate or repress the expression of downstream target genes, which play important roles in the regulation of cell cycle arrest, DNA damage response and repair, apoptosis, and senescence, to maintain cellular homeostasis^8–11^. p53, as a transcription factor, specifically recognizes and directly binds to consensus DNA sequences to activate or repress the transcription of downstream target genes, such as *MDM2*^12^, *p21*^13^, *NOXA*^14^, *RPS27L*^15,16^, and *PUMA*^17^. The canonical p53-binding motif RRRC(A/T)(A/T)GYYY(N)_0-13_RRRC(A/T)(A/T)GYYY (where R represents A or G, Y represents C or T, and N represents any nucleotide) is usually located near the transcription start site^10^. Accumulating evidence has shown that p53 induces the transcription of several upstream inhibitors of mTORC1, including PTEN, TSC2, and REDD1, resulting in the suppression of mTORC1 activity, which is frequently found to be increased in multiple cancers^18,19^. Thus, it is intriguing to explore the underlying correlation between p53 and mTORC2 activity via DEPTOR, a component and natural inhibitor of the mTORC1 and mTORC2 complexes.

In this study, we demonstrated that p53 directly binds to the *DEPTOR* promoter and activates its transcription. p53-mediated DEPTOR expression suppressed cell proliferation and survival by inhibiting AKT activity in unstressed conditions. In addition, activation of p53 by genotoxic agents (e.g., doxorubicin) significantly enhanced DEPTOR expression and induced cell resistance to doxorubicin by alleviating the feedback inhibition from S6K1 to IRS1, to activate AKT. Together, we revealed a novel mechanism by which p53 regulates cell proliferation, survival, and chemosensitivity by directly transactivating DEPTOR expression.

## Results

### DEPTOR expression is dependent on the presence of p53 in cancer cells and mouse tissues

**Fig. 1:**
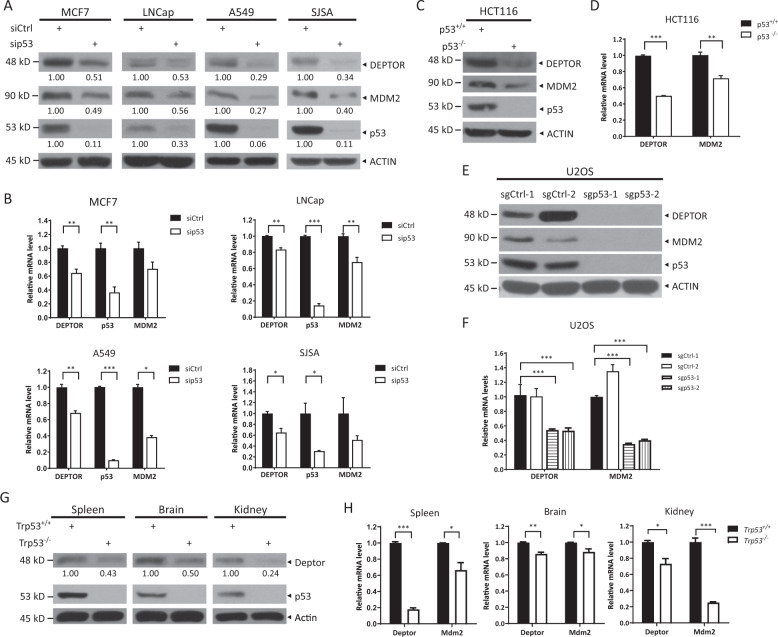
p53 positively regulates the expression of DEPTOR under unstressed conditions. **a**, **b** Silencing of p53 decreases the expression of DEPTOR at both protein **a** and mRNA levels **b**. Multiple cancer cell lines harboring wild-type p53 were transfected with siRNA targeting p53 or control scrambled siRNA for 48 h and then subjected to immunoblotting (IB) with the indicated antibodies (Abs) **a** or quantitative reverse transcription (qRT-PCR) analysis **b** (mean ± S.E.M, *n* = 3; *p < 0.05, **p < 0.01, ***p < 0.001). **c**–**f** p53 knockout reduces the expression of DEPTOR at both protein **c**, **e** and mRNA levels **d**, **f**. HCT116 and U2OS cells, with or without p53 deletion, were harvested for IB with the indicated Abs **c**, **e** or qRT-PCR analysis **d**, **f** (mean ± S.E.M, *n* = 3; **p < 0.01, ***p < 0.001). **g**, **h** DEPTOR expression is decreased at both the protein **g** and mRNA levels **h** in multiple tissues from *Trp53*^*−/−*^ mice. The indicated tissues from littermates were homogenized and subjected to IB with the indicated Abs **g** or qRT-PCR analysis **h** (mean ± S.E.M, *n* = 3; *p < 0.05, **p < 0.01, ***p < 0.001). The band density was quantified and expressed as fold change compared with the corresponding control by setting the control value as 1 **a**, **g**.

p53 has an important role in the regulation of mTORC1 activity through the induction of PTEN, TSC2, and REDD1^18,19^. However, it is unclear whether p53 can regulate the activity of both mTORC1 and mTORC2 by targeting DEPTOR expression. To explore the interplay between p53 and DEPTOR, we first examined the protein levels of DEPTOR in multiple cancer cell lines with distinct p53 statuses whose transcriptional activity was confirmed by determining the basal and induced levels of endogenous *MDM2* and *p21*, two well-established p53 target genes, upon exposure to ionizing radiation (IR) to activate p53 (Figure S1A and S1B). We found that breast cancer cells (MCF7 and ZR75-1) and prostate cancer cells (LNCap) harboring wild-type p53 had higher levels of DEPTOR, whereas other cancer cells harboring mutant p53, including SK-BR3, MDA-MB-231, and DU145 and p53-null cells, such as PC3, had lower or undetectable levels of DEPTOR, indicating a positive correlation between DEPTOR protein levels and p53 activity (Figure S1C). To determine a causal relationship, we silenced the expression of p53 via siRNA oligonucleotides and observed a significant decrease in the protein levels of DEPTOR and MDM2, serving as a positive control, in all the tested cancer cell lines harboring wild-type p53 (Fig. 1a). Notably, the mRNA levels of *DEPTOR* and *MDM2* were downregulated correspondingly to their respective protein levels upon p53 silencing (Fig. 1b). Consistently, both the protein and mRNA levels of DEPTOR were decreased in HCT116 *p53*^–/–^ colon cancer cells compared with those in paired HCT116 *p53*^+/+^ cells (Fig. 1c, d). To further demonstrate the positive correlation between DEPTOR expression and p53 activity, we depleted p53 by CRISPR-Cas9 technology in U2OS osteosarcoma cells harboring wild-type p53 and found an obvious decrease in the protein and mRNA levels of DEPTOR in two individual p53-null clones (Fig. 1e, f). Together, these results suggested that p53 positively regulates DEPTOR expression.

More importantly, we found that the protein and mRNA levels of Deptor were lower in the spleen, brain, and kidney from *Trp53*^–/–^ mice than in those from *Trp53*^+/+^ littermates (Fig. 1g, h), indicating that p53 may regulate DEPTOR expression in vivo under physiological conditions.

### p53 directly activates *DEPTOR* transcription

**Fig. 2:**
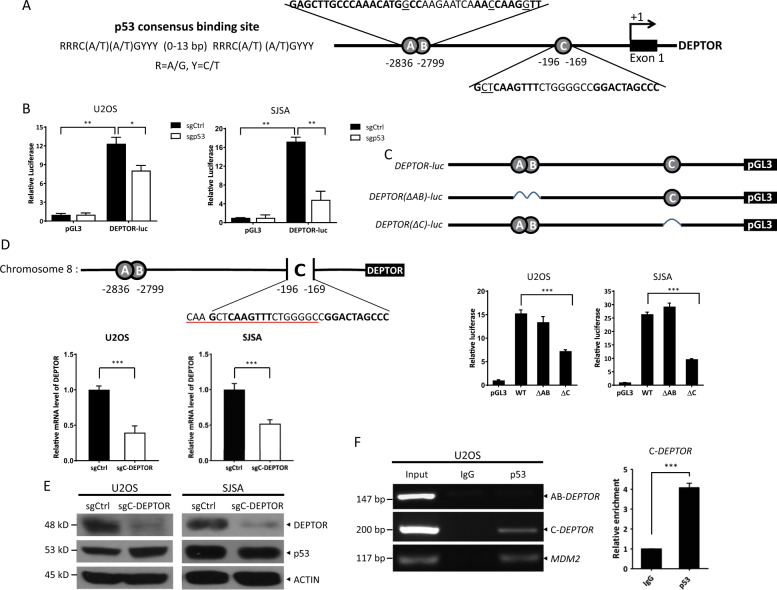
p53 directly binds to the *DEPTOR* promoter and transactivates its transcription. **a** The three putative p53-binding sites in the *DEPTOR* promoter. According to the p53 consensus DNA-binding sequence (left), three putative p53-binding sites, with mismatches underlined, were identified upstream of the “start” codon of DEPTOR (right). **b** p53 is required for the activity of *DEPTOR* promoter. Cells with or without p53 deletion were co-transfected with plasmids expressing *Renilla* luciferase and pGL3 or pGL3 containing the *DEPTOR* promoter (*DEPTOR-luc*) with three putative p53-binding sites. Luciferase activity was assessed using a luciferase assay kit and normalized to *Renilla* luciferase activity (mean ± S.E.M, *n* = 3; *p < 0.05, **p < 0.01). **c** p53-binding site C is required for the activity of the *DEPTOR* promoter. Cells were co-transfected with plasmids expressing *Renilla* luciferase and pGL3 or pGL3 containing the *DEPTOR* promoter with all the three putative p53-binding sites (WT), with the deletion of sites A and B (∆AB), or with the deletion of site C (∆C), followed by luciferase reporter assay (mean ± S.E.M, *n* = 3; ***p < 0.001). **d**, **e** p53-binding site C is required for DEPTOR expression. U2OS and SJSA cells with site C of the *DEPTOR* promoter on chromosome 8 deleted using CRISPR-Cas9 technology (sgRNA underlined), were harvested for qRT-PCR analysis **d** or IB with the indicated Abs **e**. (mean ± S.E.M, *n* = 3; ***p < 0.001) **d**. **f** p53 directly binds to site C in vivo. U2OS cells were harvested for ChIP analysis. Input samples or samples precipitated with IgG or p53 antibody were amplified using primers specific for putative p53-binding sites A, B, or C on the *DEPTOR* promoter or for the p53-binding region on the *MDM2* promoter (positive control) **f**, left, and relative enrichment of p53 at the putative p53-binding site C was quantified by qRT-PCR analysis (mean ± S.E.M, *n* = 3; ***p < 0.001) **f**, right.

We then investigated whether p53 directly activates the transcription of *DEPTOR*. First, we performed bioinformatics analysis of the *DEPTOR* promoter and identified three putative p53 consensus binding sites at –2836 ~–2817 (A-*DEPTOR*), –2826 ~–2799 (B-*DEPTOR*), and –196 ~–169 (C-*DEPTOR*), located upstream of the “start” codon of *DEPTOR* (Fig. 2a). Then, using a dual-luciferase reporter assay, we found that, compared to the pGL3 control, the activity of the luciferase reporter driven by the *DEPTOR* promoter (*DEPTOR-luc*), containing all of the three putative p53 consensus binding sites, was increased more than 10-fold in both U2OS and SJSA cells with wild-type p53, whereas *DEPTOR-luc* activity was strongly suppressed upon p53 depletion (Fig. 2b), indicating p53-dependent transcriptional activation of *DEPTOR*. To determine the exact p53-binding site in the *DEPTOR* promoter, we further constructed two luciferase reporters with the deletion of putative p53-binding sites (∆AB and ∆C). Results showed an obvious decrease in the activity of the luciferase reporter without site C (Fig. 2c), suggesting that the putative p53-binding site C, but not sites A and B, is important for the activity of the *DEPTOR* promoter. Moreover, we used CRISPR-Cas9 technology to delete site C from the *DEPTOR* promoter on chromosome 8, without disturbing its “start” codon, to examine whether the putative p53-binding site C controls DEPTOR expression under physiological conditions. Indeed, both the mRNA and protein levels of DEPTOR were significantly downregulated when site C was deleted in both U2OS and SJSA cells (∆C-*DEPTOR*), indicating that site C may play a role in p53-mediated *DEPTOR* transcription (Fig. 2d, e). More importantly, we performed chromatin immunoprecipitation (ChIP) assays and detected a physical interaction between the p53 protein and site C of the *DEPTOR* promoter, but no interaction between p53 and sites A or B. The *MDM2* promoter, containing a well-established p53-binding site, was used as a positive control (Fig. 2f, left). And relative fourfold enrichment of the p53-binding site C on *DEPTOR* promoter was quantified by qRT-PCR analysis (Fig. 2f, right). Taken together, these results demonstrated that p53 directly binds to site C of the *DEPTOR* promoter (–196 ~–169) and activates its transcription.

### p53 inhibits cell proliferation, survival, and cell cycle progression via the induction of DEPTOR

**Fig. 3:**
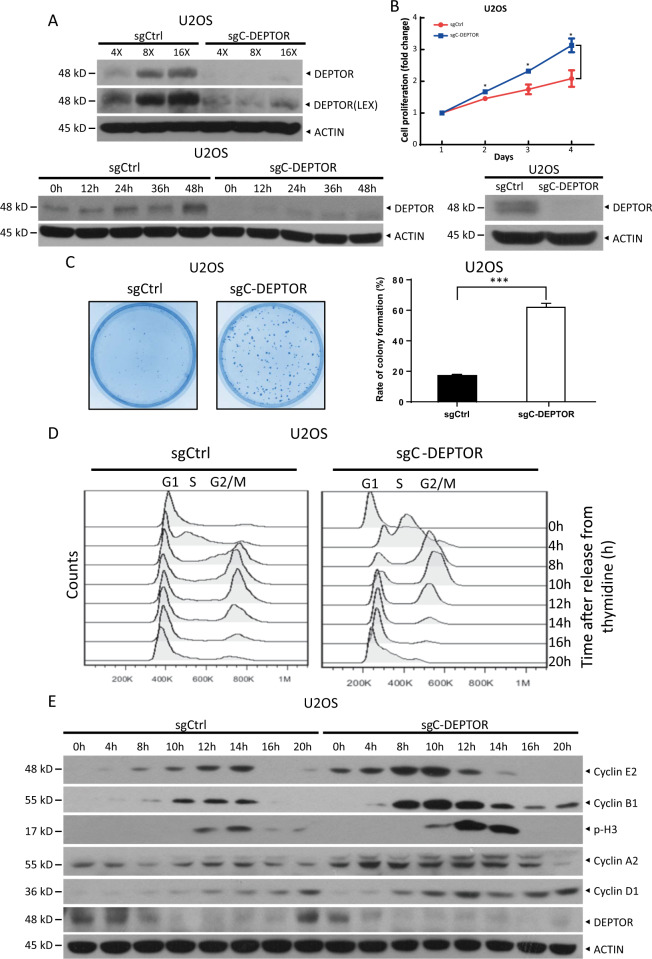
p53-mediated DEPTOR expression suppresses cell proliferation, survival, and cell cycle progression. **a** Deletion of p53-binding site C on the *DEPTOR* promoter abrogates the increase in DEPTOR expression in a cell density-dependent manner. U2OS cells, with or without the C site, were seeded at different concentrations (4 × 10^5^, 8 × 10^5^, and 16 × 10^5^) in 100 mm dishes and grown for 24 h (top) or seeded at the same density and grown for the indicated time periods (bottom). Cells were then harvested and subjected to IB with the indicated Abs. **b** Deletion of p53-binding site C on the *DEPTOR* promoter accelerates cell proliferation. Cells with or without site C of the *DEPTOR* promoter were seeded in triplicate in 96-well plates and grown for various time periods, followed by ATPlite-based cell proliferation assay. Cell proliferation is expressed as the fold change compared with day 1 (mean ± S.E.M, *n* = 3; *p < 0.05). **c** Deletion of p53-binding site C on the *DEPTOR* promoter promotes clonogenic survival. Cells with or without site C of the *DEPTOR* promoter were seeded in triplicate in 60 mm dishes at 300 cells per dish and incubated for 14 days, followed by staining (left) and colony counting (right) (mean ± S.E.M, *n* = 3; ***p < 0.001). **d**, **e** Deletion of p53**-**binding site C on the *DEPTOR* promoter accelerates cell cycle progression. Cells were treated with 2 mM thymidine for 14 h and then released for 9 h. Next, cells were treated with thymidine for an additional 14 h and then incubated in fresh medium for different time periods. Cells were then harvested for flow cytometry **d** or IB with the indicated Abs **e**.

Next, we evaluated the biological consequences of disturbing p53-mediated DEPTOR expression. The protein levels of DEPTOR were increased more dramatically in HCT116 *p53*^+/+^ cells than in HCT116 *p53*^−/−^ cells when grown to a higher density by seeding a great number of cells (Figure S2A) or by increasing the culture time with the same number of cells (Figure S2B). Consistently, the cell density-dependent increase in DEPTOR protein levels was nearly completely abrogated upon depletion of site C in the *DEPTOR* promoter (∆C-*DEPTOR*) (Fig. 3a). Given that DEPTOR is a natural inhibitor of mTOR, which regulates cell proliferation and survival, we hypothesized that cells at a higher confluency express higher levels of DEPTOR transcribed by p53 to suppress mTOR activity and prevent cell overgrowth, as a negative feedback signal to inhibit cell proliferation.

We then performed ATPlite and colony formation assays to examine whether p53-mediated DEPTOR expression regulates cell proliferation and survival. Indeed, depletion of site C (∆C-*DEPTOR*) significantly promoted cell proliferation (Fig. 3b) and colony formation (Fig. 3c), along with the suppression of DEPTOR expression (Fig. 3b), indicating that DEPTOR expression induced by p53 may suppress cell proliferation and survival. Moreover, we performed a rescue experiment to exclude the possibility of off-target effects due to CRISPR-Cas9 technology. We reconstituted ∆C-*DEPTOR* cells with ectopically expressed DEPTOR by infecting with a retrovirus expressing FLAG-DEPTOR (Figure S3B) or transfecting with FLAG-tagged DEPTOR plasmid (Figure S3E) and found that both stable and transient expression of exogenous DEPTOR significantly reversed the promotion of cell proliferation (Figure S3A and S3D) and survival (Figure S3C and S3F) induced by depletion of *DEPTOR* site C. Finally, we synchronized U2OS WT-*DEPTOR* and ∆C-*DEPTOR* cells at the end of G1 phase by double thymidine treatment and then released them back into the cell cycle to evaluate the role of p53-mediated DEPTOR expression in cell cycle progression. Flow cytometry results showed that most of the ∆C-*DEPTOR* cells entered into the S, G2/M, and G1 phases at 4, 8, and 14 h post release (one cell cycle is ~14 h), respectively, whereas the whole-cell cycle of U2OS WT-*DEPTOR* cells was ~20 h (Fig. 3d), suggesting that the depletion of site C accelerates cell cycle progression. Immunoblotting analysis of the same cell fractions at each time point largely supported the results of flow cytometry. Specifically, an increase in the levels of cyclin E2, a marker of the G1/S phase, was observed at 4 h post release in ∆C-*DEPTOR* cells, but was delayed to 8 h in WT-*DEPTOR* cells. The levels of cyclin B1, which are significantly increased in the G2/M phase, were dramatically increased at 8 h post release in ∆C-*DEPTOR* cells, but at 10 h post release in WT-*DEPTOR* cells. Further, phosphorylation of histone H3 (p-H3), as a mitotic marker (M phase), was observed at 10 h and was diminished at 14 h post release in ∆C-*DEPTOR* cells, whereas WT-*DEPTOR* cells began to enter M phase at 12 h and some cells remained in this phase at 20 h post release (Fig. 3e). These results demonstrated that disturbing p53-mediated DEPTOR expression accelerates cell cycle progression. Collectively, these results indicated that p53 activated DEPTOR expression to inhibit cell proliferation and survival and delay cell cycle progression.

### p53-mediated *DEPTOR* transcription suppresses cell proliferation and survival by inactivating AKT signaling

**Fig. 4:**
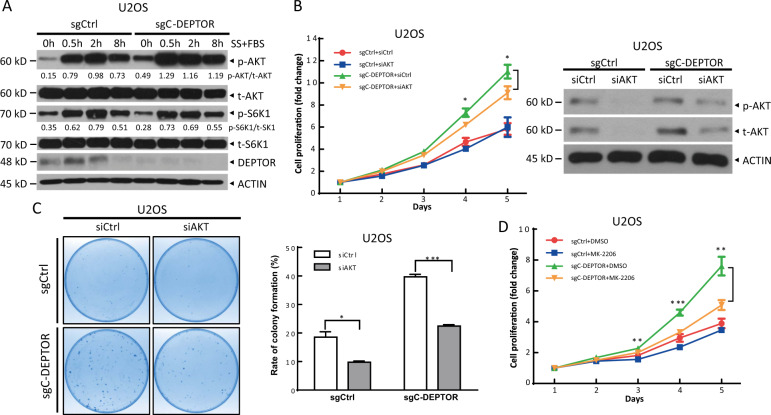
p53-mediated DEPTOR expression inhibits cell proliferation and survival via AKT inactivation. **a** Deletion of p53-binding site C on the *DEPTOR* promoter activates AKT signaling. Cells were serum-starved for 24 h, followed by serum supplementation and were then harvested at the indicated time points for IB with the indicated Abs. The band density was quantified and expressed as the ratio of p-AKT:t-AKT and the ratio of p-S6K1:t-S6K1 at various time points. **b**–**c** Silencing of AKT abrogates the induction of cell proliferation and survival by the deletion of site C. Cells with or without site C were transfected with the indicated siRNAs for 48 h, followed by ATPlite-based cell proliferation assay (**b**, left), IB with the indicated Abs (**b**, right), or clonogenic survival assay **c**. Cell proliferation is expressed as the fold change compared with day 1 (mean ± S.E.M, *n* = 3; *p < 0.05). Cell survival is expressed as the rate of colony formation (**c**, right) (mean ± S.E.M, *n* = 3; *p < 0.05, ***p < 0.001). **d** AKT inactivation abrogates the induction of cell proliferation by the deletion of site C. Cells with or without site C were seeded in triplicate in 96-well plates and then treated with MK-2206 or left untreated, followed by ATPlite-based cell proliferation assay. Cell proliferation is expressed as the fold change compared with day 1 (mean ± S.E.M, *n* = 3; **p < 0.01, ***p < 0.001).

To understand the mechanism by which p53-mediated DEPTOR expression inhibited cell proliferation and survival, we first compared the activity of mTORC1 and mTORC2 in WT-*DEPTOR* and ∆C-*DEPTOR* U2OS cells. We found that ∆C-*DEPTOR* cells exhibited higher mTORC2 activity. Specifically, after serum starvation for 24 h, the basal level of AKT phosphorylation at Ser 473, reflecting mTORC2 activity, was dramatically higher in ∆C-*DEPTOR* cells than in WT-*DEPTOR* cells (Fig. 4a, lanes 5 vs 1). After serum stimulation to activate mTORC1 and mTORC2, AKT phosphorylation at Ser 473 increased rapidly and was maintained at high levels for >8 h in ∆C-*DEPTOR* cells (Fig. 4a), indicating higher mTORC2 activity after serum stimulation upon deletion of site C in the *DEPTOR* promoter. However, the activity of mTORC1, as reflected by S6K1 phosphorylation, was not significantly different between WT-*DEPTOR* and ∆C-*DEPTOR* cells (Fig. 4a). These results suggested that p53 may induce DEPTOR expression to inhibit AKT activity, leading to the suppression of cell proliferation and survival. To confirm the causal role of AKT activation in the induction of cell proliferation and survival upon depletion of *DEPTOR* site C, we inactivated AKT using gene knockdown or small molecular inhibitors and found that AKT silencing or MK-2206 (an AKT inhibitor) treatment remarkably inhibited the proliferation of ∆C-*DEPTOR* cells, but had a minor or no effect on the proliferation of WT-*DEPTOR* cells (Fig. 4b, d). Furthermore, silencing of AKT also abrogated the increase in the survival of ∆C-*DEPTOR* cells, as reflected by decreased colony formation (Fig. 4c). Together, our results supported the notion that inhibiting p53-mediated DEPTOR expression promoted cell proliferation and survival by activating AKT.

### p53-induced DEPTOR expression suppresses cancer cell sensitivity to doxorubicin

**Fig. 5:**
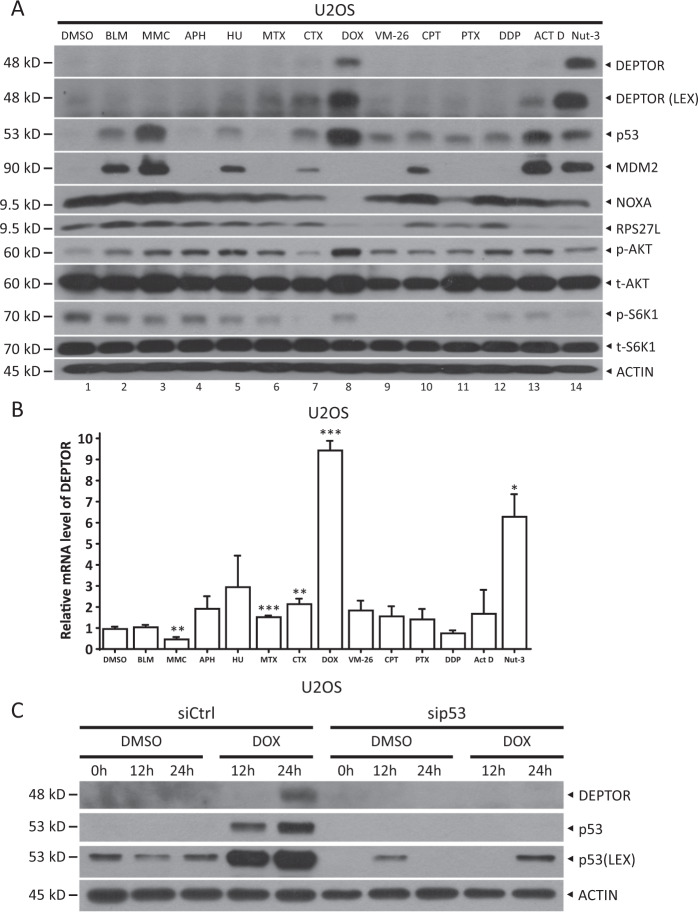
Doxorubicin treatment induces DEPTOR expression via p53. **a**, **b** Doxorubicin treatment dramatically induced DEPTOR expression at both protein and mRNA levels. U2OS cells were treated with various genotoxic agents for 24 h and then harvested for IB with the indicated Abs **a** or qRT-PCR analysis **b** (mean ± S.E.M, *n* = 3; *p < 0.05, **p < 0.01, ***p < 0.001, compared with cells treated with DMSO). *BLM* bleomycin, *MMC* mitomycin C, *APH* aphidicolin, *HU* hydroxyurea, *MTX* methotrexate, *CTX* cyclophosphamide, *DOX* doxorubicin, *VM-26* teniposide, *CPT* camptothecin, *PTX* paclitaxel, *DDP* cisplatin, *Act D* actinomycin D, and *Nut-3* nutlin-3. **c** The induction of DEPTOR upon doxorubicin treatment was dependent on p53. U2OS cells were transfected with the indicated siRNAs for 48 h and then treated with doxorubicin (1 μM) for the indicated time periods, followed by IB with the indicated Abs. *LEX* longer exposure.

Given that p53 is strikingly activated in response to several stresses, especially DNA damage, we then determined whether p53 promotes DEPTOR expression upon treatment with different chemotherapeutic agents that trigger DNA damage. Indeed, p53 was activated by several genotoxic agents, including bleomycin, mitomycin C, aphidicolin, hydroxyurea, methotrexate, cyclophosphamide, doxorubicin, teniposide, camptothecin, paclitaxel, cisplatin, and actinomycin D, as well as nutlin-3, an agent that interrupts the interaction between MDM2 and p53 to stabilize p53, in both U2OS (Fig. 5a) and SJSA (Figure S4A) cells. However, DEPTOR expression was dramatically enhanced at both protein (Fig. 5a and S4A) and mRNA (Fig. 5b and S4B) levels only by doxorubicin or nutlin-3 treatment. Next, we silenced p53 to examine whether doxorubicin treatment increased DEPTOR levels by activating p53. Indeed, siRNA-mediated knockdown of p53 by two different targeting sequences abrogated the doxorubicin-induced increase in DEPTOR expression (Fig. 5c, S4C and S4D), suggesting that doxorubicin induces DEPTOR expression in a p53-dependent manner. Moreover, siRNA-mediated p53 knockdown also suppressed DEPTOR expression induced by nutlin-3, indicating that nutlin-3 induces DEPTOR expression in a p53-dependent manner as well (Figure S4E).

**Fig. 6:**
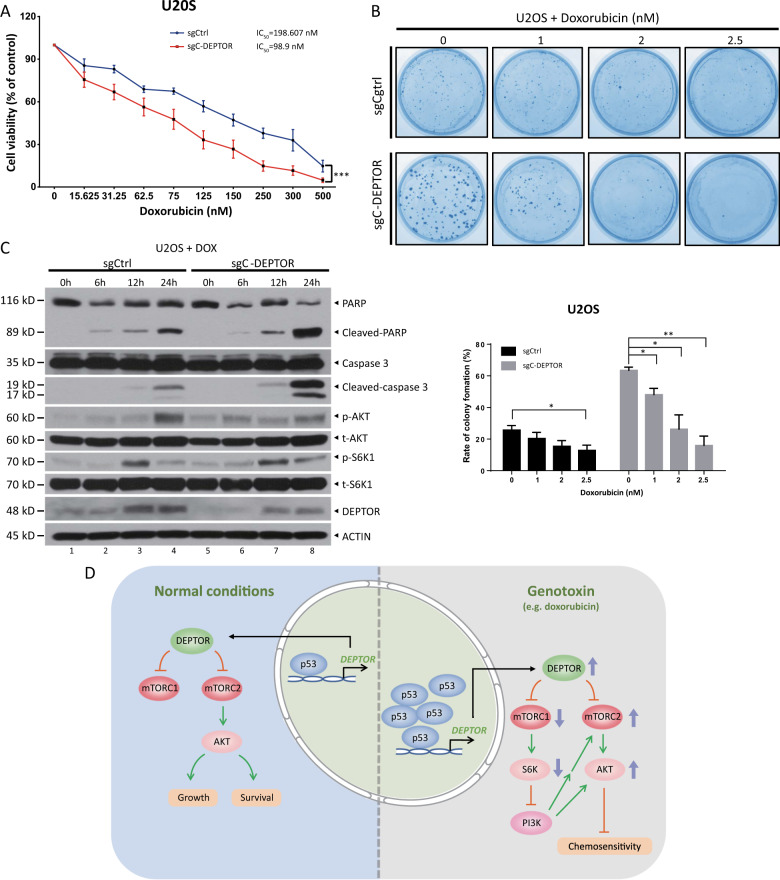
p53-induced DEPTOR expression suppresses cancer cell sensitivity to doxorubicin. **a**, **b** Deletion of p53-binding site C in the *DEPTOR* promoter sensitized cells to doxorubicin. U2OS cells with or without site C were treated with various concentrations of doxorubicin for 72 h and then subjected to ATPlite assay **a** or clonogenic survival assay **b** (mean ± S.E.M, *n* = 3; *p < 0.05, **p < 0.01, ***p < 0.001). For clonogenic survival assay, cells were seeded in triplicate in 60 mm dishes at 1000 cells (sgCtrl) or 300 cells (sgC-DEPTOR) per dish. **c** Deletion of p53-binding site C in the *DEPTOR* promoter enhanced apoptosis induced by doxorubicin via the inactivation of AKT signaling. Cells with or without site C were treated with doxorubicin (1 μM) and harvested at the indicated time points for IB with the indicated Abs. **d** A model of p53-mediated DEPTOR expression for the suppression of cell proliferation, survival, and chemosensitivity. Refer to the main text for details.

It is well known that, as an inhibitor of topoisomerase II, doxorubicin is a wide-spectrum antitumor chemotherapeutic agent that induces DNA damage and apoptosis^20^. Hence, we next attempted to determine whether p53-mediated DEPTOR expression affects cancer cell sensitivity to doxorubicin. Indeed, U2OS ∆C-*DEPTOR* cells were more sensitive to doxorubicin, with an IC_50_ of 98.9 nM, which was nearly half of the IC_50_ value for U2OS WT-*DEPTOR* cells (IC_50_ = 198.6 nM, Fig. 6a). Likewise, depletion of site C in the *DEPTOR* promoter sensitized cells to low doses of doxorubicin, as reflected by the more dramatic reduction of colony formation upon doxorubicin treatment in ∆C-*DEPTOR* cells (Fig. 6b). Mechanistically, disruption of p53-mediated DEPTOR expression significantly increased doxorubicin-induced apoptosis in a time-dependent manner, as evidenced by increased cleavage of PARP and caspase-3 in ∆C-*DEPTOR* cells (Fig. 6c). Besides, although the basal levels of AKT phosphorylation were increased (lanes 5 vs 1) upon *DEPTOR-*C site depletion, doxorubicin treatment caused a minor induction of AKT phosphorylation (lanes 8 vs 5) in ∆C-*DEPTOR* cells, but a significant induction of AKT phosphorylation in WT-*DEPTOR* cells (lanes 4 vs 1), by the relief of feedback inhibition to PI3K (Fig. 6c), which may explain the different doxorubicin sensitivities of these cells. Taken together, our results supported the notion that p53, activated by doxorubicin treatment, induced DEPTOR expression to activate AKT, thus leading to drug resistance.

## Discussion

Over the past few years, DEPTOR, a natural inhibitor of mTOR, has been shown to be an important regulator of various physiological and pathological processes, including cell growth and survival, metabolism, immunity, adipogenesis, and tumorigenesis^7^. Here, we identified and characterized DEPTOR as a novel, direct downstream target of the tumor suppressor, p53, based on the following lines of evidence: (1) the protein and mRNA levels of DEPTOR were positively correlated with p53 activity in both cultured cancer cells and mouse tissues; (2) a typical p53 consensus binding site (–196 ~–169, C-*DEPTOR*) was identified in the *DEPTOR* promoter; and (3) p53 bound to C-*DEPTOR* and activated *DEPTOR* transcription, as determined by dual-luciferase reporter and ChIP assays. Interestingly, deletion of site C moderately reduced the activity of *DEPTOR* promoter in sgp53 cells, suggesting that site C may also be regulated by other unknown transcription factor(s) besides p53, which warrants future investigations (Figure S1D). Notably, several specific factors have been identified that likely regulate *DEPTOR* transcription in cell- and context-specific manners. These factors include: (1) Glucocorticoids, which induce DEPTOR expression in a glucocorticoid receptor-dependent manner during adipogenesis^21^; (2) Baf60c-Six4, which promotes *DEPTOR* transcription to improve glucose homeostasis in skeletal muscles^22^; and (3) NOTCH1, which activates *DEPTOR* transcription in T-cell leukemogenesis^23^. However, in our study, we revealed the transcriptional regulation of DEPTOR by the tumor suppressor, p53, in various types of cancer cells, including breast cancer (MCF7), prostate cancer (LNCap), lung cancer (A549), colon cancer (HCT116), and osteosarcoma (U2OS and SJSA) cells, as well as in mouse tissues under unstressed conditions in vivo (Fig. 1 and S1), indicating a key role of p53 in regulating DEPTOR-mediated physiological processes.

Given that DEPTOR is an inhibitor of both mTOR complexes, we found that p53 regulates mTOR-mediated processes by transactivating DEPTOR expression. We performed knockout of the p53-binding site C in the *DEPTOR* promoter (∆C-*DEPTOR*) in U2OS cells harboring wild-type p53, using CRISPR-Cas9 technology and observed that: (1) ∆C-*DEPTOR* cells proliferate more rapidly than WT-*DEPTOR* cells (Fig. 3b), (2) ∆C-*DEPTOR* cells have greater survival ability (Fig. 3c), and (3) cell cycle progression is accelerated in ∆C-*DEPTOR* cells (Fig. 3d, e). Mechanistically, disturbing p53-targeted DEPTOR expression activated AKT to promote cell growth and survival (Fig. 4), suggesting that p53 may suppress mTOR signaling by transactivating DEPTOR expression, leading to the inhibition of cell proliferation and survival. Consistently, several upstream negative regulators of mTORC1, including PTEN, TSC2, AMPKβ1, and REDD1, are induced by p53 upon DNA damage^19,24–26^. In this study, we demonstrated that p53 repressed mTORC2 activity, as reflected by the decrease in phosphorylation of AKT at Ser 473, a well-known mTORC2 substrate (Fig. 4a), by inducing DEPTOR expression. Thus, our finding that p53-induced DEPTOR expression to regulate mTORC2 activity added another layer of complexity to the crosstalk between p53 and mTOR pathways.

The tumor suppressor, p53, whose transcriptional activity is significantly increased upon various stresses, has a key role in coordinating DNA damage responses^27^. In this study, we demonstrated that p53 also activates DEPTOR expression in response to genotoxic treatment. We observed that, although various genotoxic agents obviously increased the protein levels of p53, only doxorubicin had significant effects on DEPTOR expression (Fig. 5a, b, S4A and S4B). Likewise, the expression of the three well-established p53 downstream targets, MDM2^12^, NOXA^14^, and RPS27L^15,16^, was not significantly induced by most of the genotoxic agents (Fig. 5a and S4A). These results may be owing to post-translational modifications of p53, such as acetylation, which have been shown to play important roles in transactivating different p53 downstream target genes^28,29^. Thus, it would be interesting to explore whether and how doxorubicin induces the acetylation of p53 at specific lysine residue(s) and in turn, induces the p53-mediated increase in DEPTOR expression. In addition, we found that p53 upregulated DEPTOR expression in a cell density-dependent manner, whereas the protein levels of p53 were not increased (Figure S2). Although the mechanism by which p53 induces DEPTOR expression in response to cell density stress is unknown, it is worth exploring whether acetylation at specific lysine residue(s) regulates p53 activity in a cell density-dependent manner.

Given the negative feedback loop from S6K1 to IRS1/PI3K-AKT signaling, DEPTOR possesses both tumor suppressive and oncogenic properties in certain contexts^5^. In our study, we demonstrated that, by activating DEPTOR expression, p53 has distinct roles in regulating AKT activity under unstressed and genotoxic stress conditions. In unstressed cells, p53-mediated DEPTOR expression suppressed the activity of mTOR and its downstream substrate, AKT, leading to the inhibition of cell proliferation and survival (Figs. 3 and 4). However, upon doxorubicin-induced DNA damage, the dramatic induction of DEPTOR expression via p53 hyperactivation alleviated feedback inhibition from S6K1 to IRS1, thereby activating AKT, resulting in cell resistance to doxorubicin (Figs. 5 and 6). Hence, our results suggested that DEPTOR may act as a tumor suppressor at the initiation stage of tumorigenesis, when cells express normal levels of DEPTOR; however, DEPTOR, whose expression is significantly enhanced by stresses or anticancer drug treatments, exhibits oncogenic properties to promote cancer cell survival and render the cells resistant to chemotherapeutic drugs^30,31^.

In summary, our study revealed a previously unknown interplay between the tumor suppressor, p53 and the oncoprotein, mTOR, via DEPTOR. Specifically, p53 directly bound to and activated the transcription of DEPTOR, a direct inhibitor of mTOR, to inhibit cell proliferation and survival by repressing mTORC2 activity in unstressed cells. However, in response to treatment with genotoxic agents, such as doxorubicin, DEPTOR expression was dramatically enhanced by activated p53, leading to cancer cell resistance to doxorubicin by relieving feedback inhibition from S6K1 to IRS1, thus activating AKT. Therefore, DEPTOR is a novel p53 target and p53-mediated DEPTOR expression suppresses cancer cell proliferation, survival, and chemosensitivity to doxorubicin (Fig. 6d).

## Methods

### Cell lines and chemicals

U2OS, SJSA, MCF7, SK-BR3, MDA-MB-231, ZR75-1, and A549 cells were obtained from the American Type Culture Collection (ATCC) and maintained in Dulbecco’s modified Eagle’s medium (DMEM) supplemented with 10% (v/v) fetal bovine serum (FBS). HCT116 *p53*^*+/+*^ and *p53*^*−/−*^ cells were kindly provided by Professor Bert Vogelstein and maintained in McCoy’s 5 A medium containing 10% FBS. LNCap, DU145, and PC3 cells were obtained from ATCC and maintained in RPMI-1640 medium containing 10% FBS. The following chemicals were obtained from commercial sources: bleomycin (HY-17565; MCE, Monmouth Junction, NJ, USA), mitomycin C (HY-13316; MCE), hydroxyurea (HY-B0313; MCE), methotrexate (HY-14519; MCE), cyclophosphamide (HY-17420; MCE), teniposide (HY-13761; MCE), nutlin-3 (HY-50696; MCE), aphidicolin (sc-201535A; Santa Cruz Biotechnology, Dallas, TX, USA), doxorubicin (S1208; Selleck, Houston, TX, USA), camptothecin (S1288; Selleck), paclitaxel (S1150; Selleck), cisplatin (P4394; Sigma-Aldrich, St Louis, MO, USA), and actinomycin D (129935; Sigma-Aldrich).

### CRISPR/Cas9-based *DEPTOR* site C and p53 knockout

Single-guide RNA (sgRNA) was subcloned into the plasmid, pSpCas9(BB)-2A-Puro (PX459). U2OS and SJSA cells were transfected with a sequence-verified CRISPR plasmid and selected with puromycin for 3 days. Single clones were picked under a microscope and confirmed by sequencing and immunoblotting. The sequences of the sgRNA were used as follows: sgC-DEPTOR: 5′-CAAGCTCAAGTTTCTGGGGC-3′; sgp53: 5′-GCAGTCACAGCACATGACGG-3′.

### siRNA silencing

Cells were transfected with the indicated siRNA oligonucleotides in 60 mm dishes using Lipofectamine 2000, according to the manufacturer’s instructions (Invitrogen, Carlsbad, CA, USA). siCtrl:5′-ATTGTATGCGATCGCAGAC-3′. sip53: 5′-CACCATCCACTACAACTACAT-3′. sip53-2: 5′GCACAGAGGAAGAGAATCT-3′. siAKT: 5′-GAGTTTGAGTACCTGAAGCTG-3′.

### ATPlite-based cell proliferation assay

Cells were seeded in triplicate in 96-well plates (2 × 10^3^ per well). Cell proliferation was evaluated by the ATPlite assay, according to the manufacturer’s instructions (PerkinElmer, Waltham, MA, USA). Results are expressed as the fold change compared with the control. To calculate IC_50_ values, cells were cultured for 3 days with the indicated concentrations of doxorubicin and then subjected to the ATPlite assay.

### Clonogenic survival assay

A total of 300 cells seeded in 60 mm dishes were grown for 14 days and then stained with Coomassie brilliant blue. The dishes were photographed for colony counting (50 or more cells in a colony). The rate of colony formation (%) was calculated as colony number/300 × 100%.

### Dual-luciferase reporter assay

Cells were co-transfected with pGL3 or pGL3 containing the DEPTOR promoter, along with a plasmid expressing *Renilla* luciferase. After 24 h, cells were harvested and lysed for the determination of luciferase activity, using a luciferase assay kit (Promega, Madison, WI, USA), according to the manufacturer’s instructions. Relative luciferase activity was normalized to *Renilla* luciferase activity.

### ChIP

ChIP was performed using the SimpleChIP® Enzymatic Chromatin IP Kit (Magnetic Beads; #9003; Cell Signaling Technology, Danvers, MA, USA), following the manufacturer’s instructions. After reverse cross-linking and DNA purification, the immunoprecipitated DNA was amplified by PCR using the following primers: DEPTOR-C-F: 5′-CGCCCAGATGTTTATATTTTCCT-3′; DEPTOR-C-R: 5′-TGCCCTCACAGACGCTTCC-3′; DEPTOR-AB-F: 5′-AATTTTACAAAAGAGGGACAGCAA-3′; DEPTOR-AB-R: 5′-CAAGGAGAAAAGGGCACTCATAT-3′; MDM2-F: 5′-GGTTGACTCAGCTTTTCCTCTTG-3′; MDM2-R: 5′-GGAAAATGCATGGTTTAAAT-3′.

### Quantitative reverse transcription PCR

Total RNA was isolated using TRIzol reagent (Invitrogen) and then reverse transcribed into cDNA using the PrimeScript™ RT reagent kit (Perfect Real Time; Takara Biotechnology, Kusatsu, Japan) following the manufacturer’s instructions. Expression levels were measured by quantitative PCR using the SYBR® Premix Ex Taq™ kit (Tli RNaseH Plus; Takara Biotechnology), according to the manufacturer’s instructions. The primers sequences were used as follows: hDEPTOR-F:5′-GCAGCAGGAATGAAGGTCTG-3′, and hDEPTOR-R:5′-GTATGTGCGGAGAAGACTCGTAT-3′ for human DEPTOR; hp53-F: 5’-TGGAGAATATTTCACCCTTCAGATC-3’, and hp53-R: 5′-TTTTTATGGCGGGAGGTAGACT-3′ for human p53; hMDM2-F: 5′-CTCTCAGATGAAGATGATGAGGTATATC-3′, and hMDM2-R: 5′-GTTTTCCAGTTTGGCTTTCTCAG-3′ for human MDM2; hp21-F: 5′-GGCAGACCAGCATGACAGATT-3′, and hp21-R: 5′-GACTAAGGCAGAAGATGTAGAGCG-3′ for human p21; hGAPDH-F: 5′-AGGGCATCCTGGGCTACAC-3′, and hGAPDH-R: 5′-GCCAAATTCGTTGTCATACCAG-3′ for human GAPDH; mDEPTOR-F:5′-GCTGCAGGGATGAAGGTCTG-3′, and mDEPTOR-R:5′-CAAACAGCGTATGAAAGACAAGGT-3′ for mouse DEPTOR; mp53-F: 5′-GAGAGTATTTCACCCTCAAGATCCG-3′, and mp53-R: 5′-CCCCACTTTCTTGACCATTGTTT-3′ for mouse p53; mMDM2-F: 5′-GATGAGGATGATGAGGTCTATCGG-3′, and mMDM2-R: 5′-TCTGGAAGCCAGTTCTCACGAA-3′ for mouse MDM2; mGAPDH-F: 5′-GCCGCCTGGAGAAACCTGCC-3′, and mGAPDH-R: 5′-GGTGGAAGAGTGGGAGTTGC-3′ for mouse GAPDH.

### Immunoblotting

Cells were harvested, lysed, and subjected to immunoblotting, as previously described^32^. About 30–60 μg of protein were loaded on sodium dodecyl sulfate polyacrylamide gel electrophoresis gels, and the bands with appropriate exposure time are presented. Antibodies against the following proteins were used: DEPTOR (11816, for human samples; Cell Signaling Technology, Danvers, MA, USA; 1:1000), DEPTOR (09-463, for mouse samples; Millipore, Burlington, MA, USA; 1:1000), p53 (32532, for mouse samples; Cell Signaling Technology; 1:1000), p-AKT (S473) (4060; Cell Signaling Technology; 1:2000), t-AKT (4691; Cell Signaling Technology; 1:1000), p-S6K1 (T389) (9234; Cell Signaling Technology; 1:1000), PARP (9532; Cell Signaling Technology; 1:1000), caspase-3 (9665; Cell Signaling Technology; 1:1000), cyclin A2 (4656; Cell Signaling Technology; 1:1000), cyclin E2 (4132; Cell Signaling Technology; 1:1000), cyclin B1 (12231; Cell Signaling Technology; 1:1000), cyclin D1 (2978; Cell Signaling Technology; 1:1000), p-H3 (3377; Cell Signaling Technology; 1:1000), t-S6K1 (sc-230; Santa Cruz Biotechnology; 1:1000), NOXA (OP180; Calbiochem, San Diego, CA, USA; 1:1000), MDM2 (OP46; Calbiochem; 1:500), p53 (OP43, for human samples; Calbiochem; 1:1000), p21 (sc-6246; Santa Cruz Biotechnology; 1:500), actin (ET1701-80; HuaAn Biotechnology, Hangzhou, China; 1:10,000).

### Flow cytometry

Cells were treated with 2 mM thymidine (T1895; Sigma-Aldrich, St Louis, MO, USA) for 14 h, followed by 9 h of release. Next, cells were treated with thymidine for another 14 h and then incubated in fresh medium for different time periods. Cells were harvested and fixed in ice-cold 70% ethanol overnight. Cells were then stained with PI staining buffer (BD Pharmingen, San Jose, CA, USA) and subjected to flow cytometry.

### Statistical analysis

Statistical analyses were performed by two-tailed Student’s *t* tests, using Statistical Program for Social Sciences software 20.0 (SPSS, Chicago, IL, USA). Data are expressed as mean ± standard error of the mean (S.E.M). The statistical significance level was set at *p* < 0.05.

## Supplementary information

Supplementary Figure Legends

Supplementary Figure 1

Supplementary Figure 2

Supplementary Figure 3

Supplementary Figure 4
