# Supplementary material for: DEPTOR is a direct p53 target that suppresses cell growth and chemosensitivity
Source: Cell Death Dis. 2020 Nov 12;11(11):976. doi: 10.1038/s41419-020-03185-3 (PMC7661726; doi:10.1038/s41419-020-03185-3)
Supplement: Supplementary file 1 — Supplementary Figure Legends [file 41419_2020_3185_MOESM1_ESM.docx]

**Supplemental figure legends**

**Figure S1. The expression of DEPTOR in multiple cancer cell lines with different p53 statuses and the p53 binding site C on the *DEPTOR* promoter**

**(a, b)** The expression of MDM2 and p21 at both protein **(a)** and mRNA levels **(b)** are increased in response to ionizing radiation (IR) exposure in cancer cells harboring wild-type p53. The cells were irradiated at 8 Gy and then subjected to IB with the indicated antibodies (Abs) at 6 h post-IR **(a)** or qRT-PCR analysis at 3 h post-IR (mean ± S.E.M, n = 3; **p* < 0.05, ***p* < 0.01, ****p* < 0.001) **(b)**. **(c)** The expression of DEPTOR in multiple cancer cell lines with different p53 statuses. Cells with different *TP53* statuses were harvested and subjected to IB with the indicated Abs. **(d)** p53 transcribes DEPTOR through the binding site C in the *DEPTOR* promoter. Cells with or without p53 deletion were co-transfected with plasmids expressing *Renilla* luciferase and pGL3 or pGL3 containing the *DEPTOR* promoter with all the three putative p53 binding sites (WT) or with the deletion of site C (∆C), followed by luciferase reporter assay (mean ± S.E.M, n = 3; ****p* < 0.001). LEX, longer exposure.

**Figure S2. p53 regulates DEPTOR expression in a cell density-dependent manner**

HCT116 cells with or without p53 were seeded at different concentrations (4 × 10^5^, 6 × 10^5^, 8 × 10^5^, and 10 × 10^5^) in 60 mm dishes and cultured for 24 h **(a)** or seeded at the same cell density and cultured for various time periods **(b)**. Cells were then harvested and subjected to IB with the indicated Abs.

**Figure S3. Ectopic expression of DEPTOR reverses the increase in cell proliferation and survival induced by the inhibition of p53-mediated DEPTOR expression**

**(a-c)** U2OS cells with or without site C were infected with a retrovirus expressing mock vector or DEPTOR and were then selected for stable expression using puromycin. Cells were seeded in triplicate in 96-well plates and were then subjected to ATPlite-based cell proliferation assay **(a)**, harvested for IB with the indicated Abs **(b)**, or seeded in triplicate in 60 mm dishes at 300 cells per dish for clonogenic survival assay **(c)** (mean ± S.E.M, n = 3; **p* < 0.05). (**d-f**) U2OS cells with or without site C were transfected with mock vector or FLAG-tagged DEPTOR for 24 h. Then, the cells were seeded in triplicate in 96-well plates, followed by ATPlite-based cell proliferation assay **(d)**, harvested for IB with the indicated Abs **(e)**, or seeded in triplicate in 60 mm dishes at 300 cells per dish for clonogenic survival assay **(f)** (mean ± S.E.M, n = 3; **p* < 0.05, ***p* < 0.01, ****p* < 0.001).

**Figure S4.** **Doxorubicin treatment increases DEPTOR expression via p53**

**(a, b)** Doxorubicin treatment dramatically increased DEPTOR expression at both protein and mRNA levels. SJSA cells were treated with the indicated DNA damage-inducing agents for 24 h, and then harvested for IB with indicated Abs **(a)** or qRT-PCR analysis **(b)** (mean ± S.E.M, n = 3; **p* < 0.05, ***p* < 0.01, ****p* < 0.001, compared to cells treated with DMSO). BLM, bleomycin; MMC, mitomycin C; APH, aphidicolin; HU, hydroxyurea; MTX, methotrexate; CTX, cyclophosphamide; DOX, doxorubicin; VM-26, teniposide; CPT, camptothecin; PTX, paclitaxel; DDP, cisplatin; Act D, actinomycin D; and Nut-3, nutlin-3. **(c, d)** The doxorubicin-induced increase in DEPTOR expression was dependent on p53. SJSA **(c)** and U2OS **(d)** cells were transfected with the indicated siRNAs for 48 h and then treated with doxorubicin (1 μM) for the indicated time periods. Cells were then harvested and subjected to IB with the indicated Abs. **(e)** The nutlin-3-induced increase in DEPTOR expression is dependent on p53. U2OS and SJSA cells were transfected with the indicated siRNAs for 48 h and then treated with nutlin-3 (20 μM) for the indicated time periods, followed by IB with the indicated Abs.
