# Supplementary figures and images for: DEPTOR is a direct p53 target that suppresses cell growth and chemosensitivity
Source: Cell Death Dis. 2020 Nov 12;11(11):976. doi: 10.1038/s41419-020-03185-3 (PMC7661726; doi:10.1038/s41419-020-03185-3)

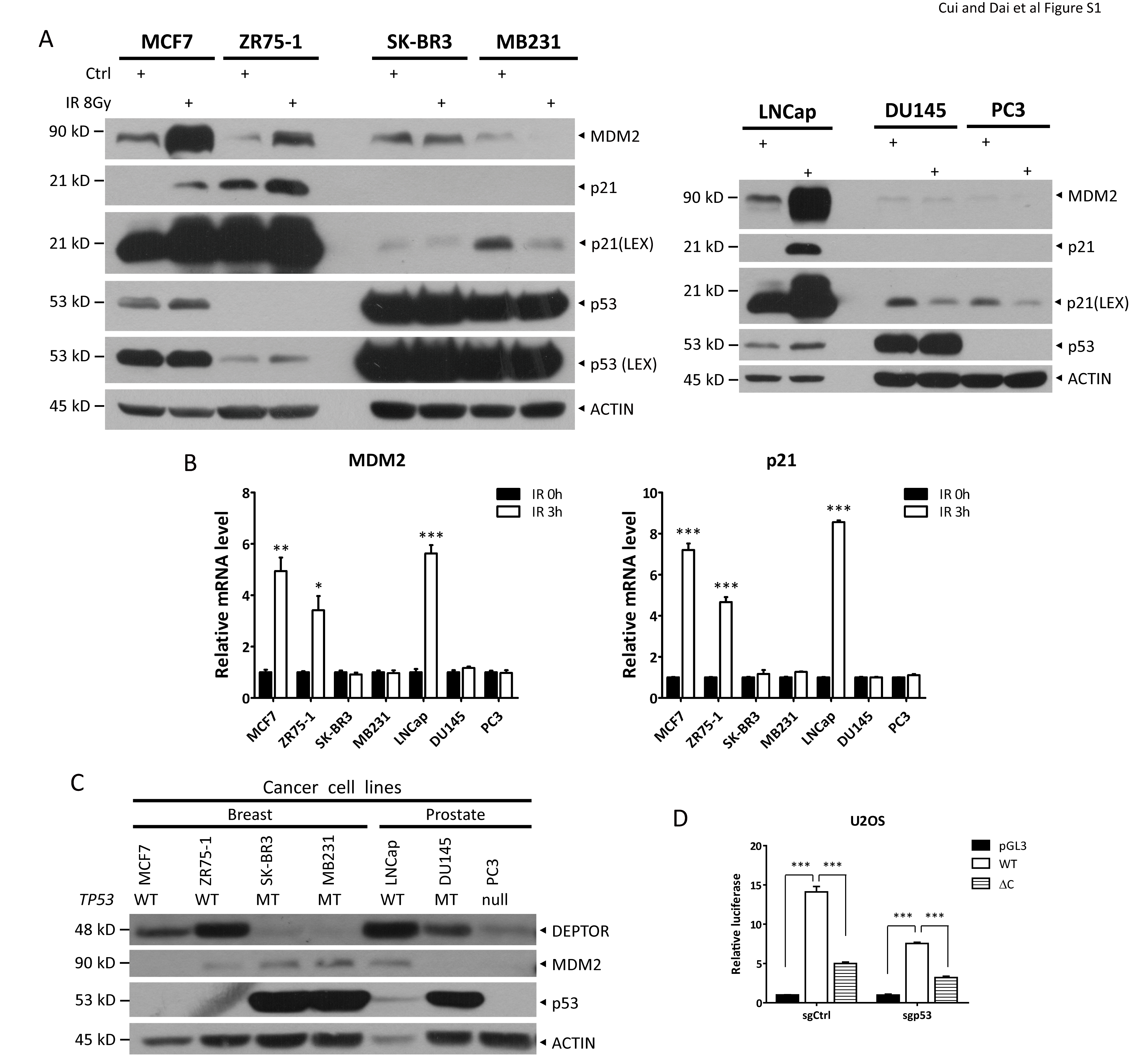

Supplement: Supplementary file 2 — Supplementary Figure 1 [file 41419_2020_3185_MOESM2_ESM.png]

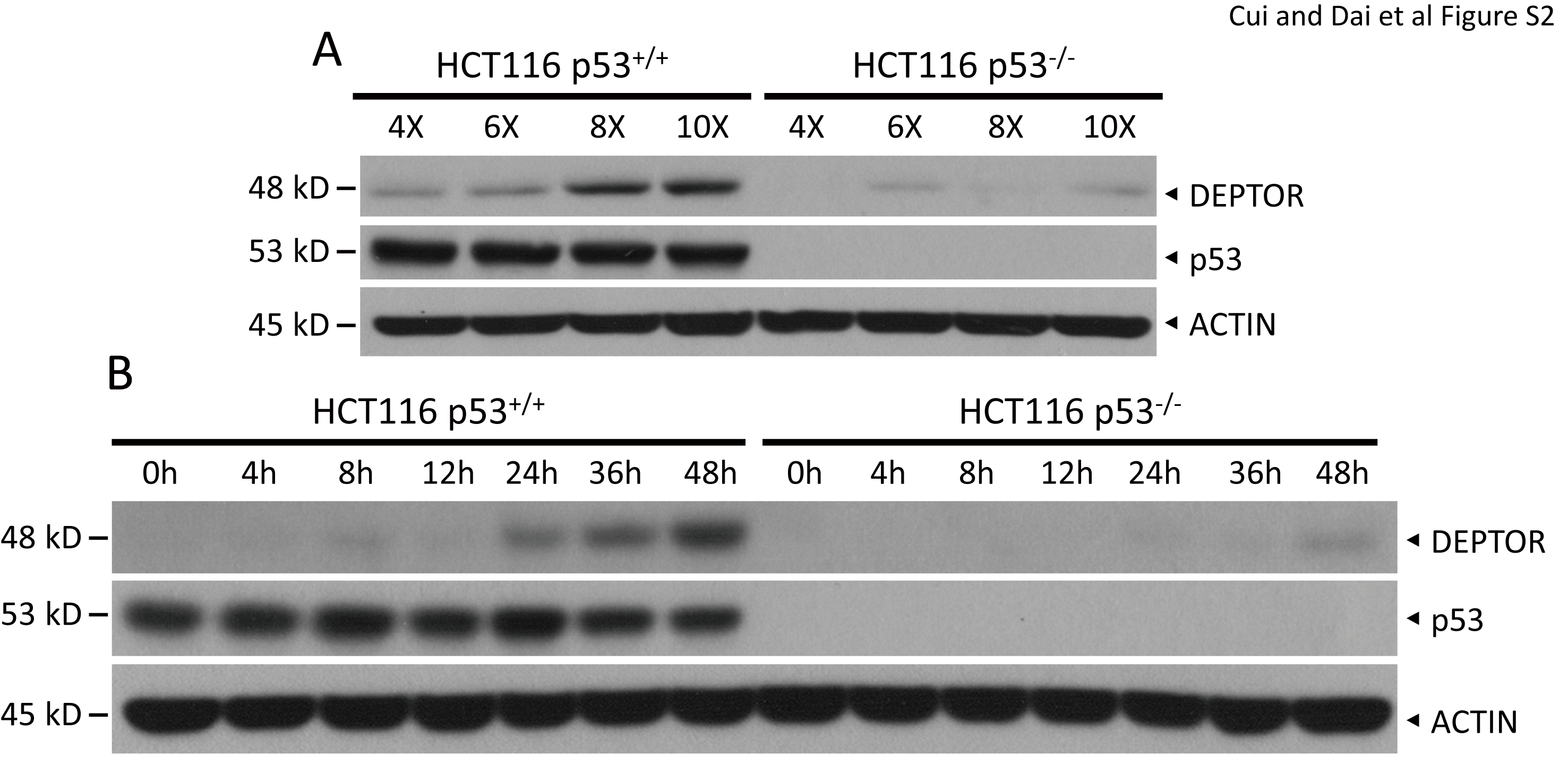

Supplement: Supplementary file 3 — Supplementary Figure 2 [file 41419_2020_3185_MOESM3_ESM.png]

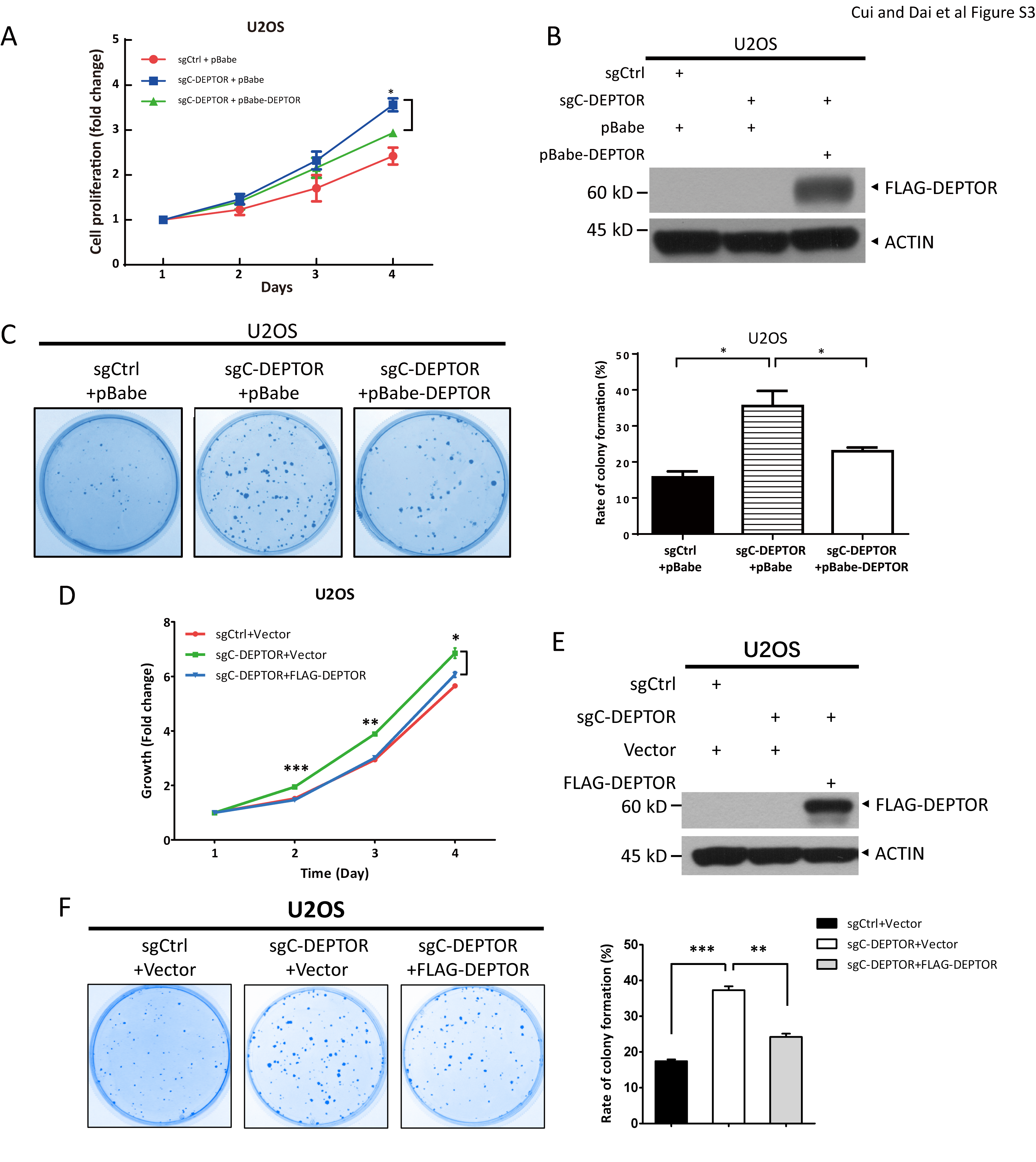

Supplement: Supplementary file 4 — Supplementary Figure 3 [file 41419_2020_3185_MOESM4_ESM.png]

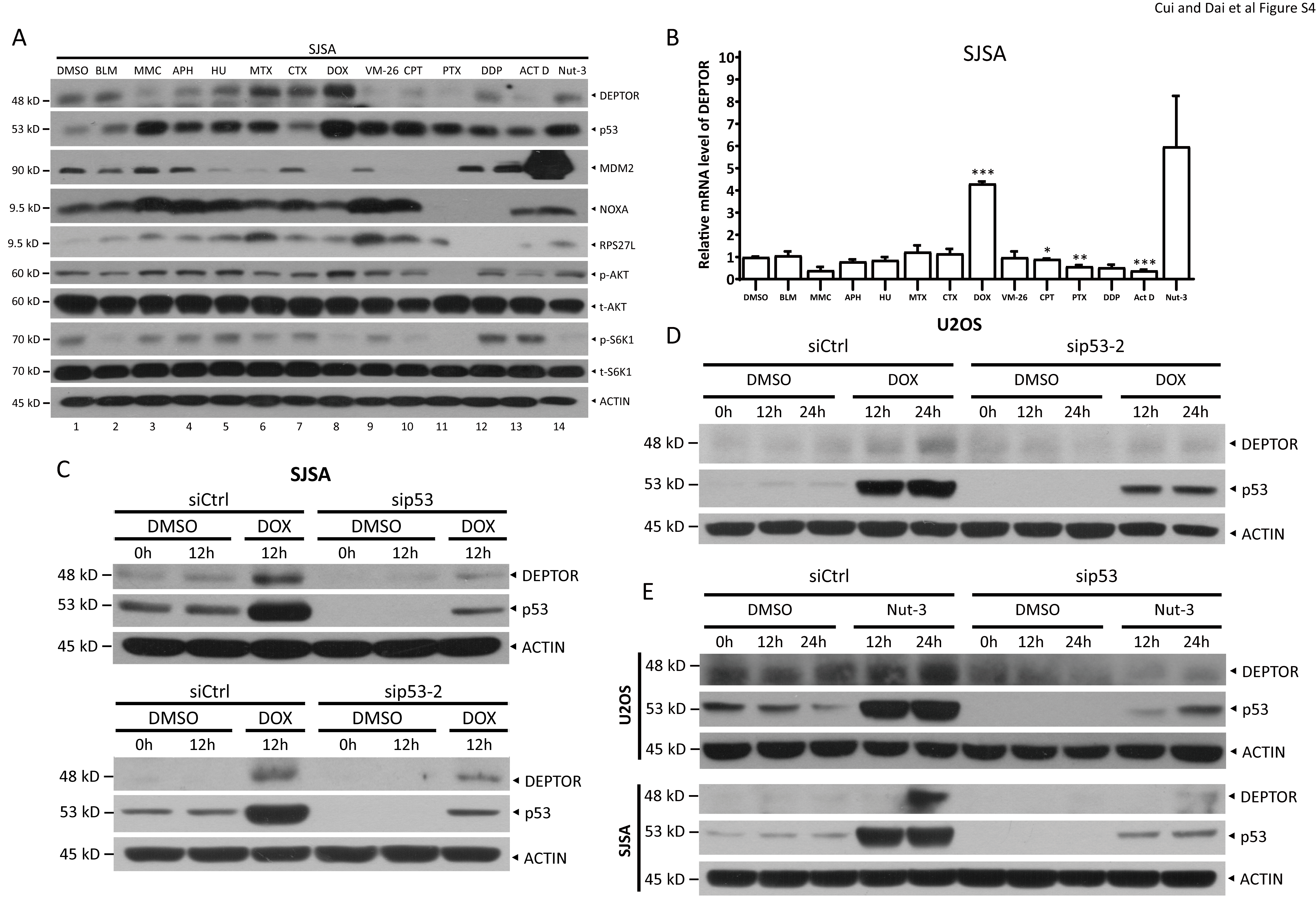

Supplement: Supplementary file 5 — Supplementary Figure 4 [file 41419_2020_3185_MOESM5_ESM.png]
